# Precise Creation of Elite Multilocular Germplasm Using a CBE NG System in *Brassica napus*

**DOI:** 10.1111/pbi.70658

**Published:** 2026-04-03

**Authors:** Huailin Li, Limin Hu, Yang Yu, Sukanta Bala, Yungu Zhai, Yang Yang, Xiaoxiao Shen, Hanzi He, Chuchuan Fan

**Affiliations:** ^1^ National Key Laboratory of Crop Genetic Improvement Huazhong Agricultural University Wuhan Hubei China; ^2^ Hubei Hongshan Laboratory Wuhan Hubei China; ^3^ Crop Research Institute, Xinjiang Academy of Agricultural Sciences China; ^4^ National Engineering Research Center of Rapeseed Huazhong Agricultural University Wuhan China

**Keywords:** base editing, *BnaCLV3*, *Brassica napus*, cytidine deaminase, multilocular silique

Dear Editor,

1

Multilocular silique is a desirable agricultural trait that has great potential in developing high‐yield *Brassica* crops due to increased seeds per silique and enhanced shatter resistance (Fan et al. 2014). A few multilocular lines of *Brassica* crops have been identified in nature, such as the multilocular ‘Yellow Sarson’ in *B. rapa* and ‘Duoshi’ in *B. juncea* (Liu 2000). The trait is conferred by recessive nuclear genes. In *B. rapa* , a causal C‐to‐T nucleotide substitution in a *CLAVATA3* (*CLV3*) homologue, causing a Pro9‐to‐Leu change in the CLE motif, confers the multilocular phenotype (Fan et al. 2014), accompanied by a significant improvement in plant yield (Figure S1). However, no stable natural multilocular germplasm has been identified in the important oilseed species *B. napus* , hindering breeding.

Previously, we pioneered the application of CRISPR/Cas9 for generating knockout mutants of *BnaCLV3* homologues in *B. napus* (Yang et al. 2018). The resulting double homozygous mutation of *BnA04.CLV3* and *BnC04.CLV3* (*a* ^*n*^ *a* ^*n*^ *c* ^*n*^ *c* ^*n*^) developed multilocular siliques with more seeds than wild‐type (WT). However, these lines exhibited pleiotropic developmental defects (excessive leaves, flattened stems, enlarged inflorescence apices, distorted siliques), limiting breeding utility (Figure 1). The specific amino acid substitution in *B. rapa* likely offers a better strategy. Cytidine base editors (CBEs), enabling precise C‐to‐T conversions without double‐strand breaks, provide a promising tool for creating optimised germplasm (Hu et al. 2020; Wu et al. 2020).

**FIGURE 1:**
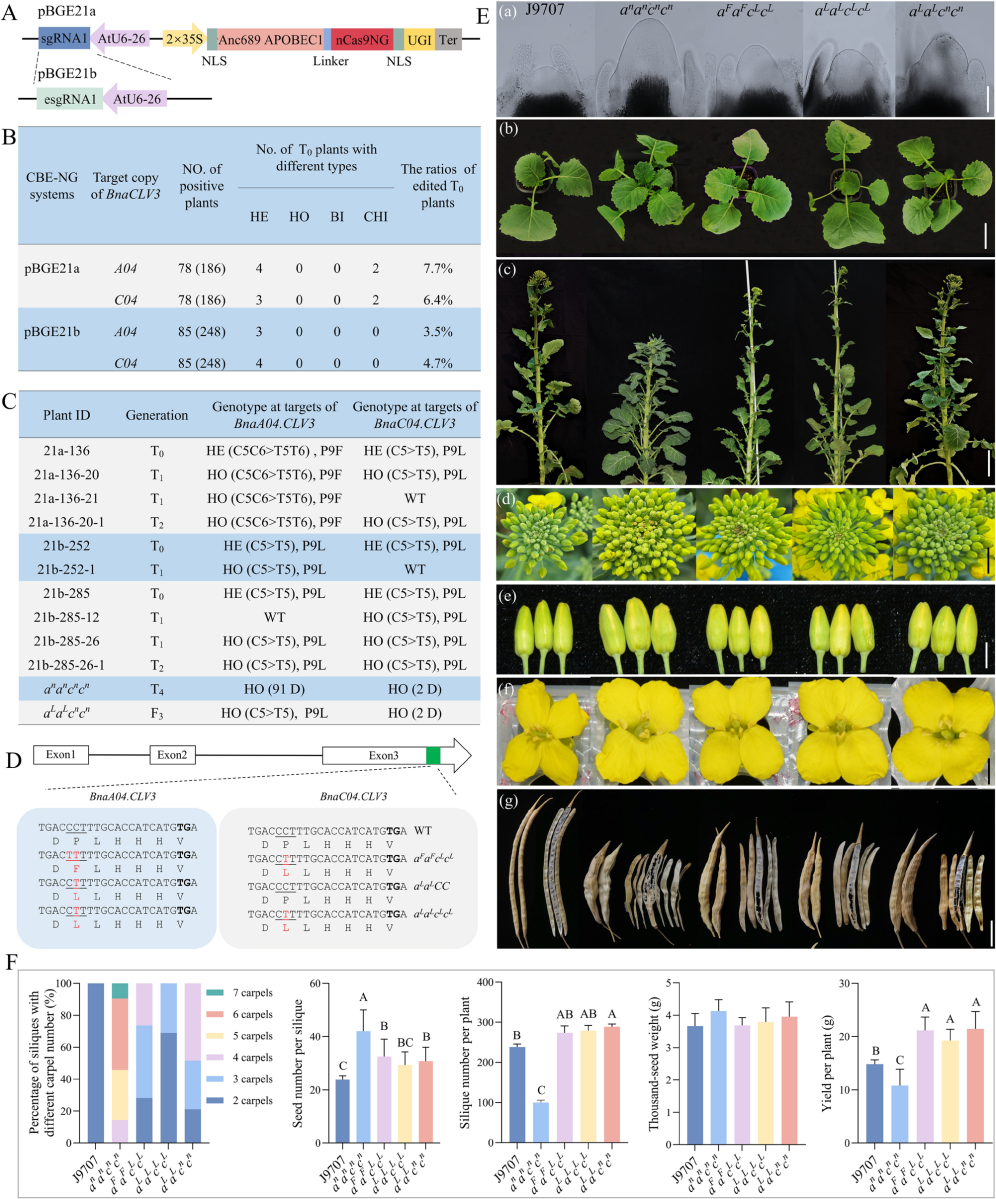
Development and application of a CBE‐Cas9NG editing system for generating elite multilocular germplasm in *Brassica napus* . (A) Constructs pBGE21a (sgRNA) and pBGE21b (esgRNA). (B) Numbers of T_0_ plants with different mutation types (homozygous, HO; heterozygous, HE; biallelic, BI; chimeric, CHI). (C) Genotypes of base‐edited (*a* ^*L*^ *a* ^*L*^ *c* ^*L*^ *c* ^*L*^, *a* ^*F*^ *a* ^*F*^ *c* ^*L*^ *c* ^*L*^), knockout (*a* ^*n*^ *a* ^*n*^ *c* ^*n*^ *c* ^*n*^) and pyramided (*a* ^*L*^ *a* ^*L*^ *c* ^*n*^ *c* ^*n*^) mutants (D, Deletion). (D) Mutation details for three base‐edited lines. PAM and targeted changes in bold/red. *a* ^*L*^ *a* ^*L*^/*a* ^*F*^ *a* ^*F*^: Homozygous Pro‐to‐Leu/Phe in *BnaA04.CLV3*; *c* ^*L*^ *c* ^*L*^/*CC*: Homozygous Pro‐to‐Leu/no mutation in *BnaC04.CLV3*. (E) Phenotypes of mutants (*a* ^*L*^ *a* ^*L*^ *c* ^*L*^ *c* ^*L*^, *a* ^*F*^ *a* ^*F*^ *c* ^*L*^ *c* ^*L*^ and *a* ^*L*^ *a* ^*L*^ *c* ^*n*^ *c* ^*n*^) compared to WT (J9707): (a) shoot apical meristem of 8‐d old seedlings, (b) leaf number of 30‐day old seedlings, (c) plant morphology at the pre‐blooming stage, (d) primary inflorescence, (e) bud, (f) flower and (g) mature silique. Scale bars: 200 μm (a), 5 cm (b), 15 cm (c), 2.5 cm (d), 5 mm (e, f), 2 cm (g). (F) Yield Traits of mutants (*a* ^*L*^ *a* ^*L*^ *c* ^*L*^ *c* ^*L*^, *a* ^*F*^ *a* ^*F*^ *c* ^*L*^ *c* ^*L*^ and *a* ^*L*^ *a* ^*L*^ *c* ^*n*^ *c* ^*n*^) compared to WT (J9707): Percentage of siliques with different carpel number, seed number per silique, silique number per plant, thousand‐seed weight, and yield per plant. The data and error bars represent the mea*n* ± SD (*n* ≥ 15 plants for each genotype). Upper‐case letters indicate a significant difference at the 0.01 probability level.

To develop high‐yielding multilocular rapeseed, this study employed CBE systems to achieve precise single‐base substitution from P_CCT_ to L_CTT_ at the ninth amino acid of the BnaCLV3 CLE domain. However, the target site lacked available NGG‐PAM. We therefore engineered a CBE‐nCas9 system (CBE‐Cas9NG) recognising NG‐PAM. The base editor (Anc689 APOBEC, nCas9NG, and UGI) from Anc689BE4max‐nCas9NG (Wang et al. 2019) replaced the Cas9 in the pYLCRISPR/Cas9P35S‐H (Yang et al. 2018), creating pBGE21. A universal sgRNA1 was designed for targeting both *BnaCLV3* copies was cloned with AtU6‐26‐driven sgRNA or enhanced sgRNA (esgRNA) cassettes into pBGE21, yielding pBGE21a/b (Figure 1A).

The vectors were introduced into J9707 via *Agrobacterium*‐mediated hypocotyl transformation, yielding 163 positive transgenic seedlings (Figure 1B). Average mutation rates of 7.05% for sgRNA and 4.10% for esgRNA indicate that the CBE‐Cas9NG system combined with the sgRNA expression cassette achieved satisfactory editing efficiency (Figure 1B). Genotyping further confirmed that not only the intended C6 to T6 (P_CCT_ to L_CTT_) substitution but also the concurrent C5C6 to T5T6 (P_CCT_ to F_TTT_) mutations were identified at the target site (Figure 1C).

T_0_ edited plants were self‐pollinated to obtain T_2_ homozygous lines: 21b‐285‐26‐1 (both *BnaCLV3* copies with Pro‐to‐Leu in CLE motif; *a* ^*L*^ *a* ^*L*^ *c* ^*L*^ *c* ^*L*^) and 21b‐136‐20‐1 (*BnaA04.CLV3* with Pro‐to‐Leu, *BnaC04.CLV3* with Pro‐to‐Leu; *a* ^*F*^ *a* ^*F*^ *c* ^*L*^ *c* ^*L*^) (Figure 1C,D). Both base‐edited lines have larger shoot apical meristems than WT (J9707), but less enlarged than the double knockout mutant *a* ^*n*^ *a* ^*n*^ *c* ^*n*^ *c* ^*n*^ (Figure 1Ea, Figure S2B). They grew normally to maturity without the severe defects seen in *a* ^*n*^ *a* ^*n*^ *c* ^*n*^ *c* ^*n*^ (Figure 1Ea–f, Figures S2–S3). Critically, both edited lines showed multilocular siliques with 2.70 and 3.14 carpels per plant on average, significantly fewer than *a* ^*n*^ *a* ^*n*^ *c* ^*n*^ *c* ^*n*^ (Figure 1Eg,F, Figures S4–S6). The average number of seeds per silique was 29.26 and 32.52 in two base‐edited lines, significantly higher than J9707 (23.83) (Figure 1F and Figure S6B). The number of siliques per plant and the thousand‐seed weight of base‐edited lines were similar to that of J9707; consequently, the yield per plant was significantly increased (by 29.7%–42.8% on average) relative to J9707 (Figure 1F). These results demonstrate that the base‐edited multilocular mutants retain normal agronomic traits and hold significant yield potential.

We further generated the homozygous line *a* ^*L*^ *a* ^*L*^ *c* ^*n*^ *c* ^*n*^ through gene pyramiding, which contains the Pro‐to‐Leu amino acid substitution in *BnaA04.CLV3* and knockout mutation in *BnaC04.CLV3* (Figure 1C and Figure S2A). Its shoot apical meristem size and leaf number were reduced compared to *a* ^*L*^ *a* ^*L*^ *c* ^*L*^ *c* ^*L*^ and *a* ^*F*^ *a* ^*F*^ *c* ^*L*^ *c* ^*L*^ mutants, but still significantly larger than the wild‐type J9707 (Figure 1Ea–b and S2B,C). It grew normally from the seedling stage to maturity (Figure 1Ec–f and Figure S3). Interestingly, the *a* ^*L*^ *a* ^*L*^ *c* ^*n*^ *c* ^*n*^ line showed multilocular siliques with an average of 3.44 carpels per plant, which was significantly higher than both *a* ^*L*^ *a* ^*L*^ *c* ^*L*^ *c* ^*L*^ and *a* ^*F*^ *a* ^*F*^ *c* ^*L*^ *c* ^*L*^ mutants (Figure 1F and Figures S4 and S5). We also evaluated the yield‐related traits of the *a* ^*L*^ *a* ^*L*^ *c* ^*n*^ *c* ^*n*^, *a* ^*L*^ *a* ^*L*^ *c* ^*L*^ *c* ^*L*^ and *a* ^*F*^ *a* ^*F*^ *c* ^*L*^ *c* ^*L*^ mutants. There were no significant differences in major agronomic traits such as seeds per silique, siliques per plant, thousand‐seed weight and yield per plant among these mutant lines. However, the yield per plant of all these lines was substantially higher than that of the J9707, whereas the *a* ^*n*^ *a* ^*n*^ *c* ^*n*^ *c* ^*n*^ mutant was significantly lower than the J9707 (Figure 1F and Figure S6). In Arabidopsis, the Pro9 in the conserved CLE motif is required for bioactivity but not receptor binding (Kondo et al. 2011). Thus, the amino acid substitutions here likely partially reduce CLV3 activity, conferring the beneficial multilocular trait without major defects.

In summary, this study developed a CBE‐Cas9NG system specifically designed for *B. napus* , which enables efficient and precise C‐to‐T base editing and substantially broadens the targeting scope for genomic modifications. This platform provides a novel tool for functional genomics and precision breeding in rapeseed and other dicotyledonous species. The novel multilocular germplasm developed in this work thus represents a promising strategy for enhancing yield in this important crop.

## Author Contributions

Chuchuan Fan designed the experiment; Huailin Li, Limin Hu and Yang Yu performed most of the experiments; Huailin Li wrote the manuscript; Chuchuan Fan revised the manuscript. All authors read and approved the final manuscript.

## Funding

This work was supported by National Key Research and Development Program of China (Grant Nos. 2022YFD1200400 and 2023YFF1000704); National Natural Science Foundation of China (Grant No. 31671279); the Technology Major Project on Key Techniques of Agricultural Biological Breeding (Grant No. 2023ZD0404203); Project of Hubei Provincial Technology Innovation (Grant No. 2024BBA001); the Key Research Projects of Hubei Province (Grant No. 2022BBA0039).

## Conflicts of Interest

The authors declare no conflicts of interest.

## Supporting information


**Figure S1:** The yield performance of natural multilocular mutants in *Brassica rapa* var. *yellow sarson*.
**Figure S2:** Creation and phenotypic characterization of the a^L^a^L^c^n^c^n^ mutant through gene pyramiding.
**Figure S3:** Whole‐Plant Phenotypic Comparison among Mutants and Wild‐Type at Maturity.
**Figure S4:** Representative transverse sections showing different carpel numbers.
**Figure S5:** Phenotypic observation of pistil and silique morphology of mutilocular rapeseed.
**Figure S6:** Phenotypic observation of mature siliques with different carpel number and their seeds per silique.

## Data Availability

The data that supports the findings of this study are available in the supporting information of this article.
