# Supplementary material for: Precise Creation of Elite Multilocular Germplasm Using a CBE NG System in Brassica napus
Source: Plant Biotechnol J. 2026 Apr 3;24(7):4716–8. doi: 10.1111/pbi.70658 (PMC13278538; doi:10.1111/pbi.70658)
Supplement: Supplementary file 1 — Figure S1: The yield performance of natural multilocular mutants in Brassica rapa var. yellow sarson. Figure S2: Creation and phenotypic characterization of the aLaLcncn mutant through gene pyramiding. Figure S3: Whole‐Plant Phenotypic Comparison among Mutants and Wild‐Type at Maturity. Figure S4: Representative transverse sections showing different carpel numbers. Figure S5: Phenotypic observation of pistil and silique morphology of mutilocular rapeseed. Figure S6: Phenotypic observation of mature siliques with different carpel number and their seeds per silique. [file PBI-24-4716-s001.docx]

**Title: Precise Creation of Elite Multilocular Germplasm Using a CBE NG System in *Brassica napus***

Huailin Li ^#^, Limin Hu ^#^, Yang Yu ^#^, Sukanta Bala, Yungu Zhai, Yang Yang, Xiaoxiao Shen, Hanzi He, Chuchuan Fan*

National Key Laboratory of Crop Genetic Improvement, Huazhong Agricultural University, Wuhan 430070, Hubei, China

Hubei Hongshan Laboratory, Wuhan 430070, Hubei, China

Crop Research Institute, Xinjiang Academy of Agricultural Sciences, Urumqi, 830091, China

National Engineering Research Center of Rapeseed, Huazhong Agricultural University, Wuhan, 430070, China

^#^ Contributed equally

*Corresponding authors: Chuchuan Fan (Tel + 86-27-87286873; Fax + 86-27-87280009; Email: [fanchuchuan@mail.hzau.edu](mailto:fanchuchuan@mail.hzau.edu).cn

**Supporting Information**

**Plant material**

The semi-winter *B. napus* pure line J9707 was used as the transformation receptor in this study. All seeds were obtained from the National Engineering Research Centre of Rapeseed, Wuhan, China. Yield-related traits were evaluated using a randomized complete block design with three biological replicate plots per genotype. Each plot consisted of six rows (1.8 m row width) with plant spacing of 15 cm × 30 cm.

**
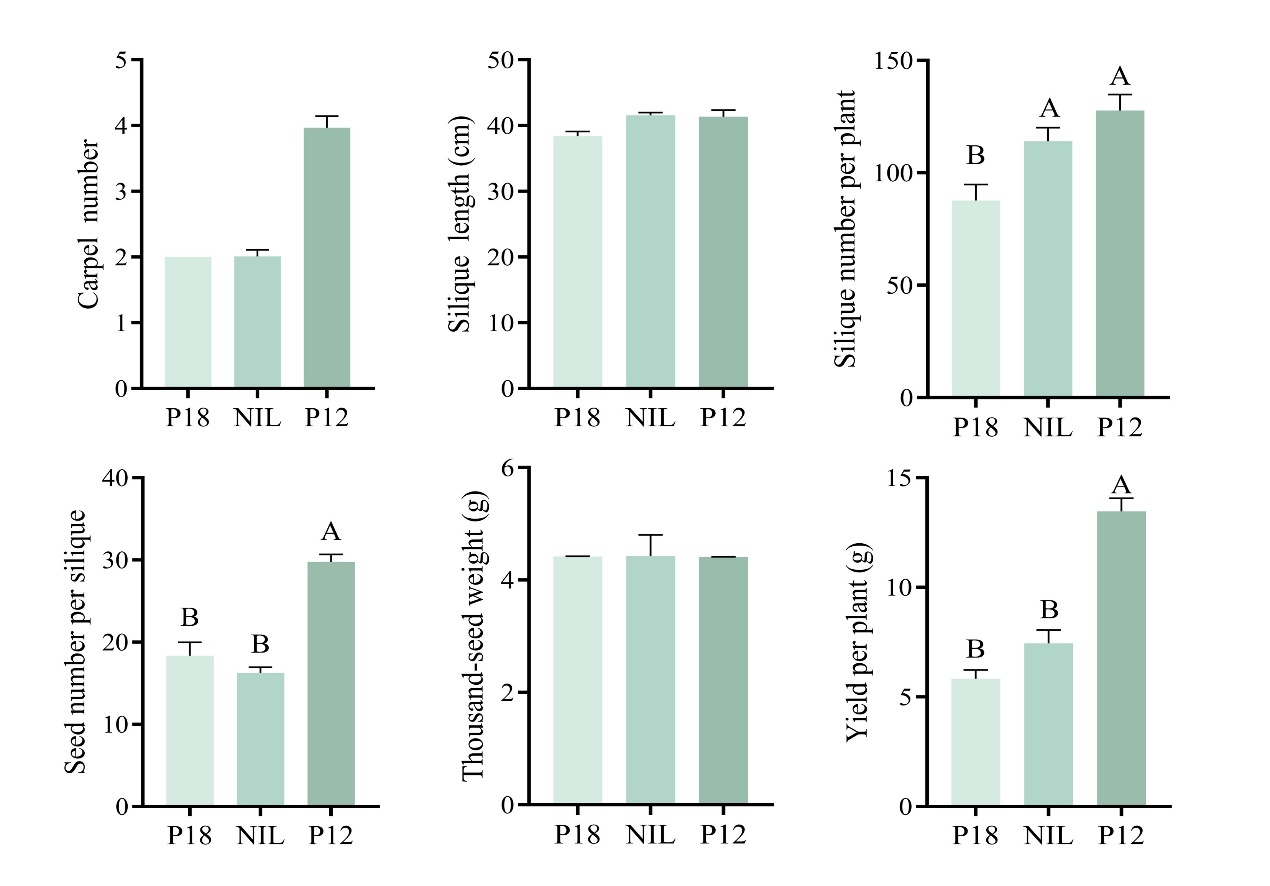
**

**Figure S1. The yield performance of natural multilocular mutants in *Brassica rapa* var. *yellow sarson.***

The carpel number, silique length, silique number per plant, seed number per silique, thousand-seed weight and yield per plant were compared among P18 (*BraCLV3*, 2 carpels), P12 (*Braclv3* mutant, 4 carpels) and P12 near-isogenic line (*BraCLV3* under P12 genetic background). The data and error bars represent the mean ± SD (*n* ≥ 15 plants for each genotype). Upper-case letters indicate a signiﬁcant difference at the 0.01 probability level.


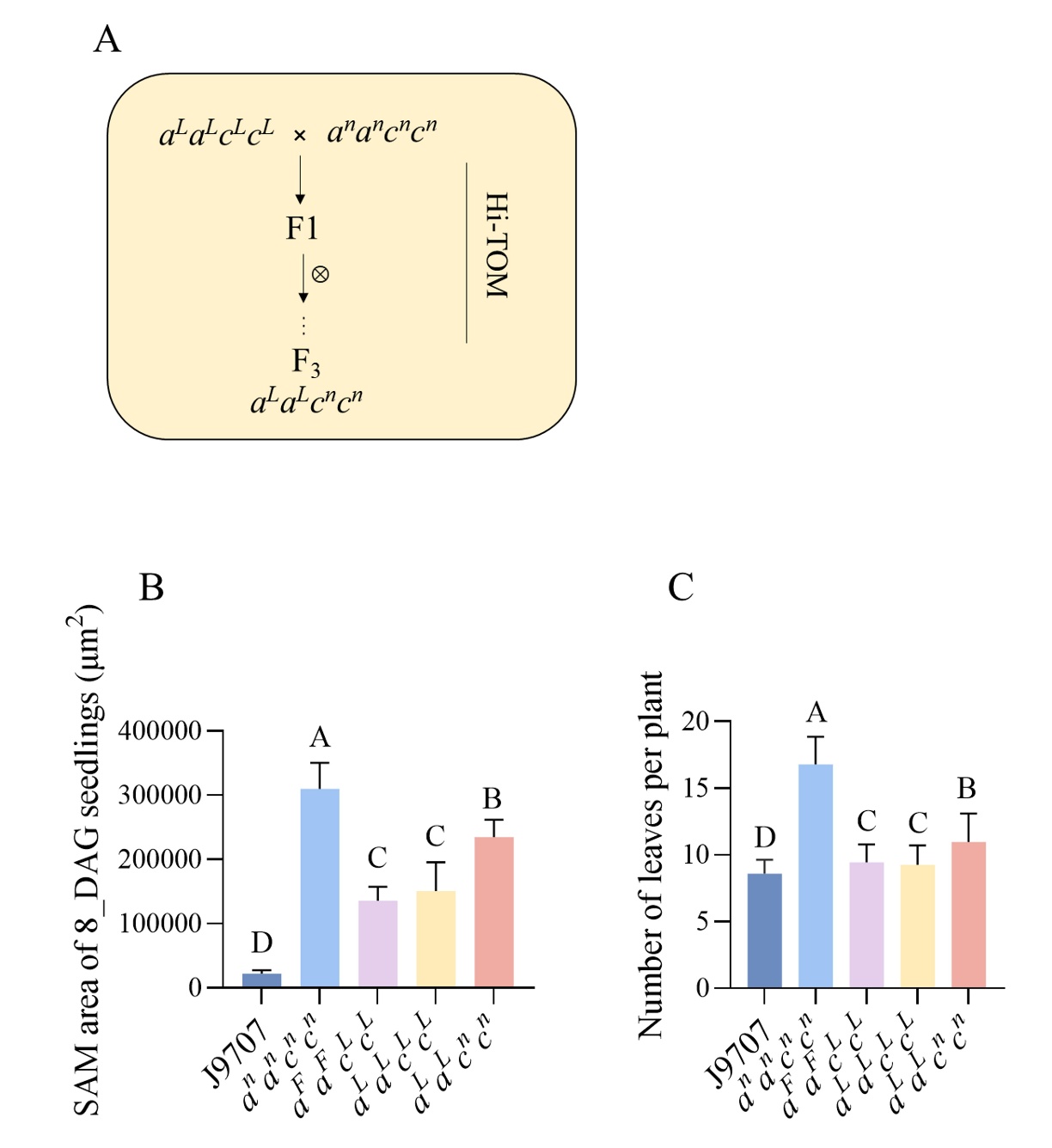


**Figure S2. Creation of the a^L^a^L^c^n^c^n^ mutant through gene pyramiding and phenotypic characterization of meristem-related traits.**

(A) Schematic illustration of the hybridization strategy used to generate the homozygous *a^L^a^L^c^n^c^n^* mutant. The homozygous *a^L^a^L^c^n^c^n^* mutant was generated by hybridizing the base-edited mutant *a^L^a^L^c^L^c^L^* and double knockout mutant *a^n^a^n^c^n^c^n^*, followed by multiple generations of self-pollination and genotyping using Hi-TOM to screen for the desired genotype. (B–C) Statistical analysis of shoot apical meristem area and leaf number in the J9707 (WT) and different homozygous *Bnaclv3* mutants. Compared to the J9707, all mutants exhibited a statistically significant increase in the shoot apical meristem area (B) and leaf number (C) at 30-d old seedlings. The double knockout mutant *a^n^a^n^c^n^c^n^* showed the most pronounced enlargement, followed by *a^L^a^L^c^n^c^n^*, while the base-edited mutants *a^L^a^L^c^L^c^L^* and *a^F^a^F^c^L^c^L^* displayed the least expansion.


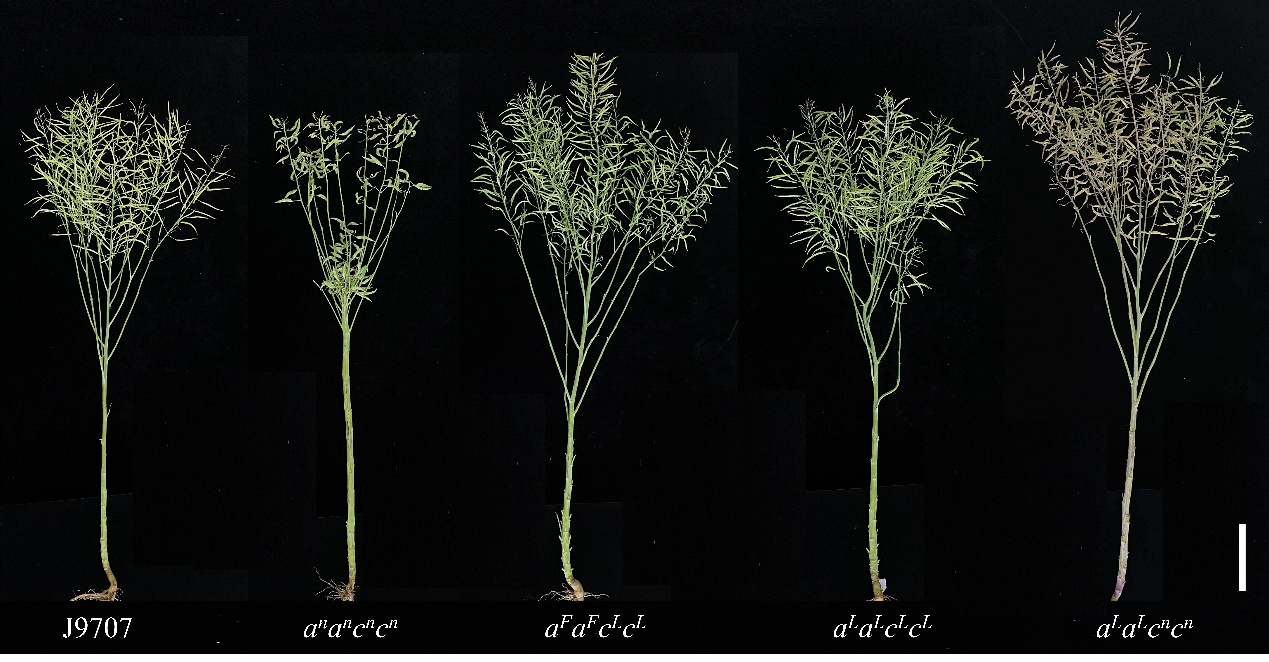


**Figure S3. Comparison of whole-plant phenotypic observation at maturity.**

The J9707(WT) and mutants of *a^F^a^F^c^L^c^L^*, *a^L^a^L^c^L^c^L^*, and *a^L^a^L^c^n^c^n^* grew normally at maturity without exhibiting significant growth defects as those observed in the double knockout mutant (*a^n^a^n^c^n^c^n^*).

**
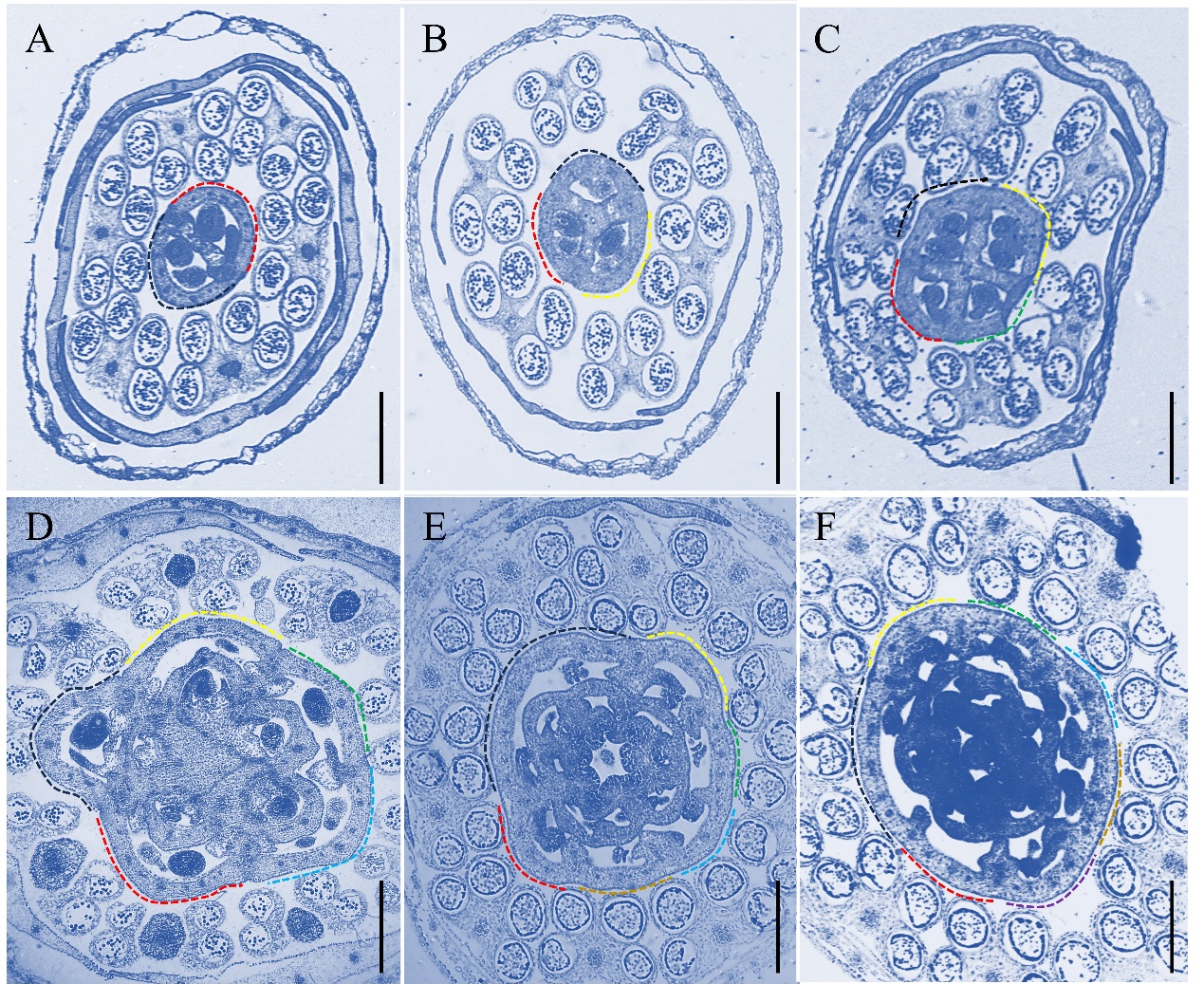
**

**Figure S4.** **Representative transverse sections showing different carpel numbers.**

(A–F) The representative transverse sections of pistils showed two (A), three (B), four (C), five (D), six (E) and seven (F) carpels, respectively. The lines of different colors in the figure are used to distinguish the various carpels. Scale bar： 0.50 mm.

**
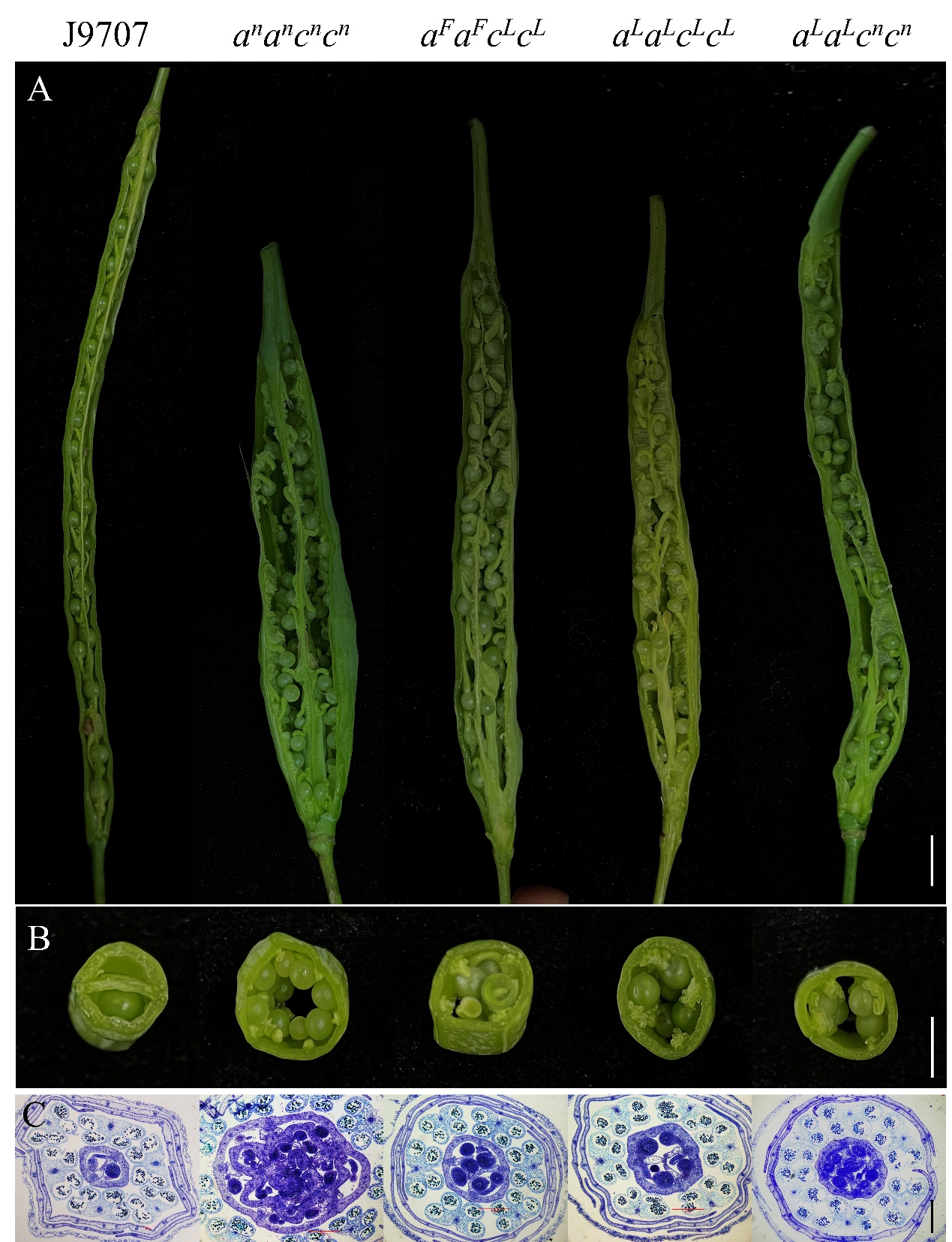
**

**Figure S5. Phenotypic observation of pistil and silique morphology of mutilocular rapeseed.**

Longitudinal (A) and cross (B) sections of representative siliques with different carpel numbers in J9707 (WT) and different mutants (*a^n^a^n^c^n^c^n^*, *a^F^a^F^c^L^c^L^*, *a^L^a^L^c^L^c^L^*, and *a^L^a^L^c^n^c^n^*), respectively; scale bar: 0.5 cm. (C) The representative cross-sections of early floral buds (paraffin-embedded) from J9707 and different mutants (*a^n^a^n^c^n^c^n^*, *a^F^a^F^c^L^c^L^*, *a^L^a^L^c^L^c^L^*, and *a^L^a^L^c^n^c^n^*), respectively; scale bar: 0.5 mm.


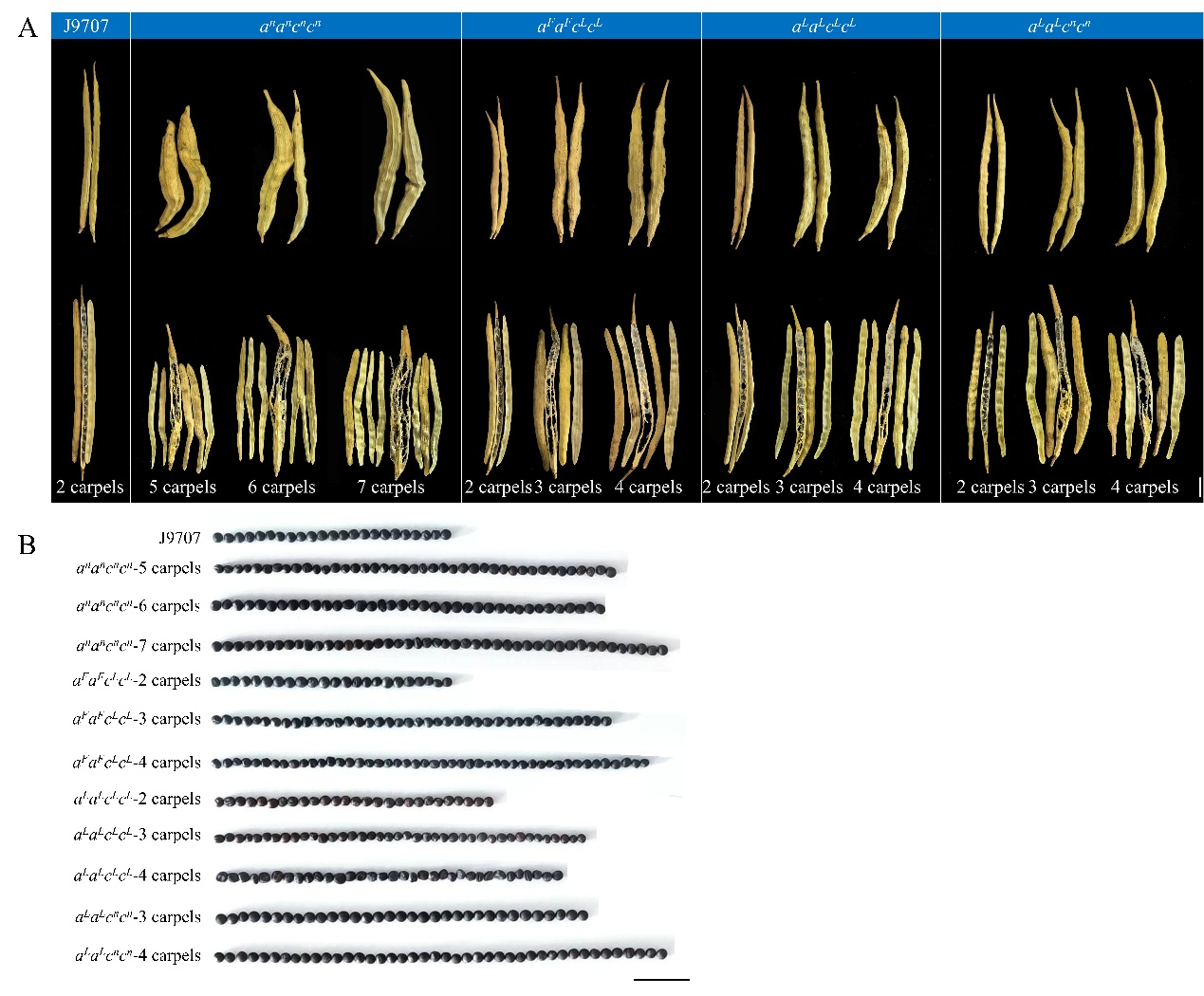


**Figure S6. Phenotypic observation of mature siliques** **with different carpel number and their seeds per silique.**

(A) Representative mature siliques with different carpel number from J9707 (WT) and different mutants (*a^n^a^n^c^n^c^n^*, *a^F^a^F^c^L^c^L^*, *a^L^a^L^c^L^c^L^*, and *a^L^a^L^c^n^c^n^*), respectively; scale bar: 1 cm. (B) Representative seed per silique in J9707 and different types of mutants. In each mutant, the representative seed number per silique is displayed separately for siliques with different numbers of carpels Scale bar: 1 cm.
